# Outdoor nighttime light exposure (light pollution) is associated with Alzheimer’s disease

**DOI:** 10.3389/fnins.2024.1378498

**Published:** 2024-09-06

**Authors:** Robin M. Voigt, Bichun Ouyang, Ali Keshavarzian

**Affiliations:** ^1^Rush Medical College, Rush Center for Integrated Microbiome and Chronobiology Research, Rush University Medical Center, Chicago, IL, United States; ^2^Department of Internal Medicine, Rush University Medical Center, Chicago, IL, United States; ^3^Department of Anatomy and Cell Biology, Rush University Medical Center, Chicago, IL, United States; ^4^Department of Neurological Sciences, Rush University Medical Center, Chicago, IL, United States; ^5^Department of Physiology, Rush University Medical Center, Chicago, IL, United States

**Keywords:** Alzheimer’s disease (AD), light pollution, artificial light at night (ALAN), Alzheimer’s disease risk factor, Alzheimer’s disease prevalence

## Abstract

**Introduction:**

Alzheimer’s disease (AD) prevalence has increased in the last century which can be attributed to increased lifespan, but environment is also important. Exposure to artificial light at night is one environmental factor that may influence AD.

**Methods:**

This study evaluated the relationship between outdoor nighttime light exposure and AD prevalence in the United States using satellite acquired outdoor nighttime light intensity and Medicare data.

**Results:**

Higher outdoor nighttime light was associated with higher prevalence of AD. While atrial fibrillation, diabetes, hyperlipidemia, hypertension, and stroke were associated more strongly with AD prevalence than nighttime light intensity, nighttime light was more strongly associated with AD prevalence than alcohol abuse, chronic kidney disease, depression, heart failure, and obesity. Startlingly, nighttime light exposure more strongly associated with AD prevalence in those under the age of 65 than any other disease factor examined.

**Discussion:**

These data suggest light exposure at night may influence AD, but additional studies are needed.

## Introduction

Throughout most of human history fire was used as the source of light (e.g., wood, tallow, wax, or oil), gas lighting emerged at the end of the 18th century, and electric lighting was developed in the mid-19th century, and by the early 1900s most homes in the United States had electric lighting (Schivelbusch, 1995; Bowers, 1998; Brox, 2010; Boyle, 2019). Nowadays artificial lights ubiquitously illuminate our indoor and outdoor spaces. Artificial outdoor lights provide safety, convenience, and aesthetics (e.g., deter crime, illuminate roadways, highlight landscaping), but excessive artificial light at night is called light pollution (e.g., poorly shielded, overly bright lighting fixtures). Today, most people living in urban and suburban areas are unable to see natural celestial light due to light pollution and as much as 80% of the global population experience light pollution. Although artificial light at night is considered by most to be harmless or even beneficial (e.g., safety), light pollution has detrimental ecological, behavioral, biological, and health consequences (Bedrosian and Nelson, 2013).

Exposure to artificial outdoor light at night is associated with numerous detrimental health effects including sleep disruption, obesity, depression, anxiety, memory dysfunction, atherosclerosis, and cancer (Bozejko et al., 2023) but little is known about the impact of light pollution on Alzheimer’s disease (AD). AD is the most common neurodegenerative disorder and accounts for 60–80% of dementia cases (Brenowitz et al., 2017; Kapasi et al., 2017) and it is estimated that 10.8% of adults over the age of 65 have AD (Rajan et al., 2021). Incidence and prevalence of AD have increased in recent decades which parallels the increase in light pollution.

To date, only a few studies have examined the impact of light at night on neurodegeneration, neuroinflammation, cognitive impairment, dementia, or AD with only two studies focused on outdoor light at night (Romeo et al., 2013; Kim et al., 2018; Walker et al., 2020; Chen et al., 2022; Delorme et al., 2022; Mazzoleni et al., 2023). These studies find that humans living in areas with brighter nighttime outdoor light in China and Italy have a higher risk of developing mild cognitive impairment and late-onset dementia (Chen et al., 2022; Mazzoleni et al., 2023). Certain groups appear to be particularly sensitive to the effects of outdoor light at night: females, those with less educational attainment, and those with a lack of social activities (Chen et al., 2022). Despite these data, the possibility that exposure to light at night may influence AD has not been carefully examined.

This study evaluated the relationship between AD prevalence and average nighttime light intensity (light pollution) in the United States (i.e., the lower 48 states) using data from the Centers for Medicare and Medicaid Services (Chronic Conditions), the Centers for Disease Control and Prevention [CDC, Behavioral Risk Factor Surveillance System (BRFSS)], and satellite-acquired light pollution data (VIIRS) (details in Supplementary material).

## Results

### Average nighttime light intensity is associated with higher prevalence of AD

**Figure 1:**
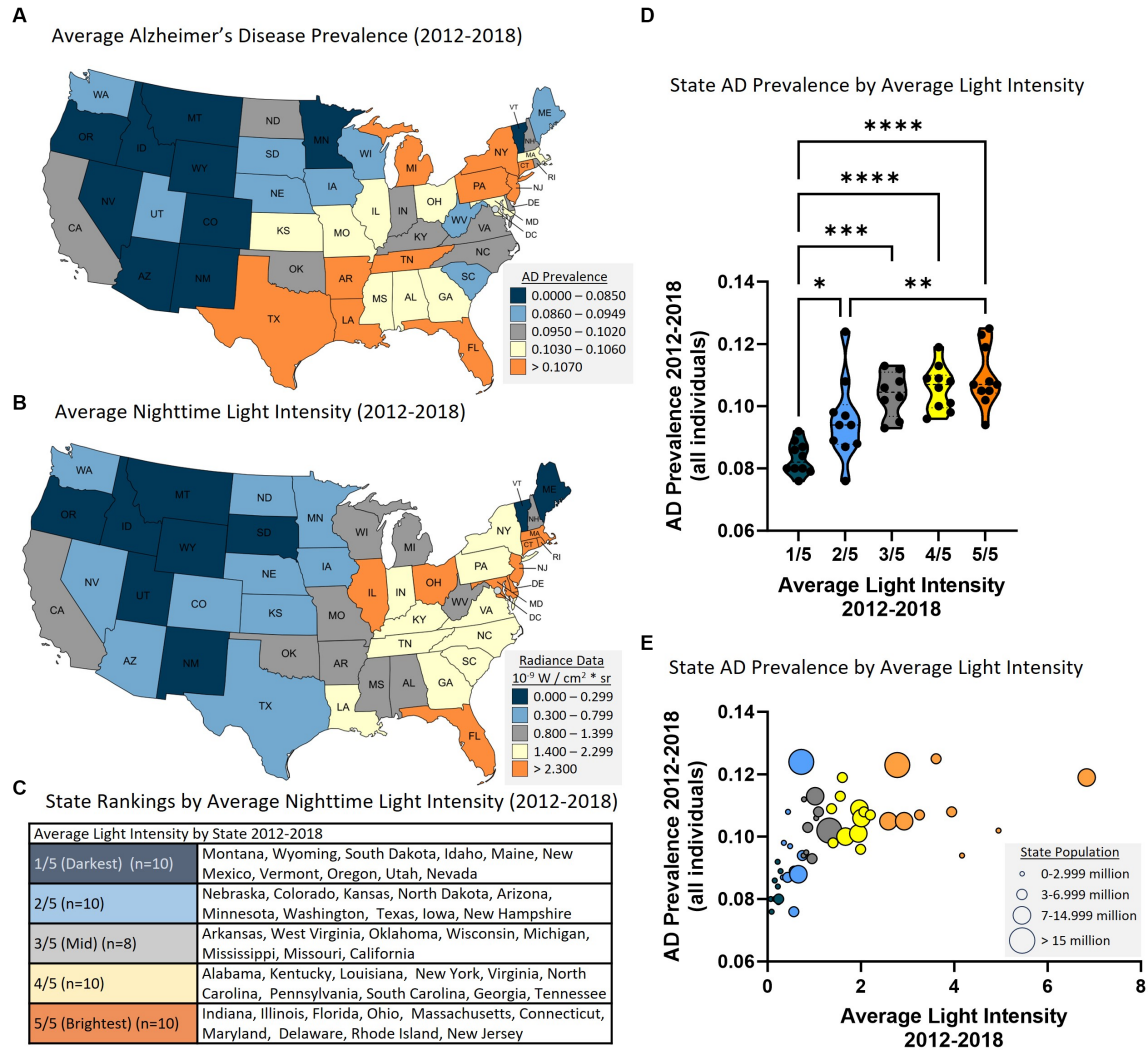
Higher state average nighttime light intensity is associated with higher state AD prevalence (2012–2018 average). **(A)** AD prevalence by state. **(B)** Average nighttime light intensity by state. **(C)** Average nighttime light intensity state rankings. 1/5 = darkest average nighttime light intensity through 5/5 = brightest average nighttime light intensity. **(D)** State AD prevalence by average state nighttime light intensity rankings. ANOVA revealed a statistically significant difference in AD prevalence between groups [*F*(4, 43) = 13.50, *p* < 0.0001]. Multiple comparisons testing found that the states with the lowest average light intensity had significantly different AD prevalence than states with higher average nighttime light intensity: 1/5 vs. 2/5 (*p* = 0.031), 1/5 vs. 3/5 (*p* < 0.001), 1/5 vs. 4/5 (*p* < 0.001), 1/5 vs. 5/5 (*p* < 0.001), and 2/5 vs. 5/5 (*p* = 0.010). ^*^p < 0.05, ^**^p < 0.01, ^***^p < 0.001, ^****^p < 0.0001. **(E)** Pearson correlation analysis between state AD prevalence and state nighttime light intensity revealed a positive relationship [*r*(46) = 0.557. *p* < 0.001]. Circle size reflects state population.

AD prevalence was acquired from Medicare data (Figure 1A) and average nighttime light intensity was generated from satellite acquired data (Figure 1B) and the data were averaged for years 2012–2018. States were ranked according to average nighttime light intensity and were divided into five groups representing those with the lowest average light intensity (i.e., 1/5, darkest) to the highest average light intensity (i.e., 5/5, brightest) (Figure 1C). Analysis revealed a statistically significant difference in AD prevalence between groups [*F*(4, 43) = 13.500, *p* < 0.001]. Multiple comparisons testing found statistical differences between states with the darkest average nighttime light intensity and states with brightest average nighttime light intensity (Figure 1D). Pearson correlation analysis similarly demonstrated a relationship between light intensity and AD prevalence [*r*(46) = 0.557, *p* < 0.001, Figure 1E]. This relationship also existed when examining those above the age of 65 [ANOVA: *F*(4, 43) = 14.560, *p* < 0.001; Correlation: *r*(46) = 0.490, *p* < 0.001] and those under the age of 65 [ANOVA: *F*(4, 43) = 6.950, *p* < 0.001; Correlation: *r*(46) = 0.550, *p* < 0.001] (Supplementary Figure S1). Each year was also assessed individually, and the same positive relationship was observed between nighttime light intensity and AD prevalence for each year independently (Supplementary Table S1).

A linear mixed model was applied to examine the overall effect of light intensity on AD prevalence (from 2012 to 2018) taking account of within-state correlation due to repeated measures. When examining all individuals, the overall regression was statistically significant (Chi-square = 1081.9, df = 1, *p* < 0.0001). Average nighttime light intensity was significantly associated with AD prevalence (regression coefficient = 0.283, SE = 0.102, 95% CI = 0.082–0.485, *p* = 0.006, *R*^2^ = 0.382, Table 1). This effect was also observed in those over the age of 65, under the age of 65, in both men and women, and in all races examined except Asian Pacific Islander (Table 1). It was noted that stronger regression coefficients were associated with certain ethnic groups compared to others (e.g., Native Americans). The factors contributing to this observation are not clear but could reflect conditions in particular geographic areas and should be investigated further.

**Table 1:** There is a significant relationship between state AD prevalence and state average nighttime light intensity from 2012 to 2018 across age, sex, and race/ethnicity.

	Regression coefficient	SE	95% CI	*p*-value	*R* ^2^
All individuals	0.283	0.102	0.082–0.485	0.006*	0.382
Age					
65+	0.328	0.125	0.082–0.575	0.009*	0.359
<65	0.211	0.060	0.094–0.329	0.001*	0.473
Sex					
Male	0.265	0.085	0.098–0.432	0.002*	0.381
Female	0.296	0.118	0.063–0.529	0.013*	0.395
Race					
Asian Pacific Islander	0.073	0.159	−0.240–0.386	**0.648**	0.174
Hispanic	0.488	0.198	0.098–0.877	0.014*	0.208
Native American	1.031	0.190	0.657–1.406	<0.001*	0.391
Non-Hispanic Black	0.671	0.182	0.313–1.030	<0.001*	0.242
Non-Hispanic White	0.306	0.101	0.107–0.504	0.003*	0.436

Linear mixed effects model data are provided for all individuals, age, sex, and race. AD prevalence was significantly associated with average nighttime light intensity in all groups except Asian Pacific Islander. SE, standard error; CI, confidence. *The bold indicate a significant *p* value.

Co-variates known (or proposed to be) risk factors for AD including alcohol abuse, atrial fibrillation, chronic kidney disease, depression, diabetes, heart failure, hyperlipidemia, hypertension, obesity, and stroke were subsequently included in the model. Data for the covariates were obtained from Medicare Chronic Conditions data or from the CDC. When looking at all individuals, average nighttime light intensity was associated with AD prevalence even when accounting for alcohol abuse, chronic kidney disease, depression, heart failure, and obesity (Table 2). These data suggest that nighttime light intensity has a stronger influence on AD prevalence than these conditions. However, other covariates were more strongly associated with AD than light intensity including atrial fibrillation, diabetes, hyperlipidemia, hypertension, and stroke (Table 2) indicating that nighttime light exposure had a more subtle effect than these disease covariates. Similar results were obtained when examining men, women, and those over the age of 65 with each having a unique profile (Table 2). However, for those under the age of 65 average light intensity was associated with AD prevalence even when considering all the covariates (estimate = 0.174, SE = 0.064, *p* = 0.007) (Table 2) suggesting that those under the age of 65 may be particularly sensitive to the effects of exposure to light at night.

**Table 2:** State average nighttime light intensity (2012–2018) is more strongly associated with State AD prevalence than some known AD risk factors.

	Regression coefficient, SEM, 95% CI, *p*-value, R^2^				
Covariate	All individuals	Male	Female	65+	<65
	65+, <65, male, female	65+ and <65	65+, <65	Male and female	Male and female
Light intensity (*Alcohol abuse*)	0.266, 0.104, 0.062–0.471, 0.011^*^, 0.364	0.240, 0.087, 0.070–0.411, 0.006^*^, 0.361	0.292, 0.119, 0.058–0.527, 0.015^*^, 0.388	0.323, 0.126, 0.075–0.571, 0.011^*^, 0.353	0.202, 0.061, 0.082–0.321, 0.001^*^, 0.462
Light intensity (*Atrial fibrillation*)	0.176, 0.098, −0.017–0.369, 0.074, 0.481	0.177, 0.084, 0.012–0.343, 0.036^*^, 0.460	0.180, 0.108, −0.033–0.392, 0.098, 0.532	0.135, 0.118, −0.097–0.367, 0.253, 0.485	0.209, 0.060, 0.090–0.328, 0.001^*^, 0.477
Light intensity (*CKD*)	0.289, 0.089, 0.113–0.465, 0.001^*^, 0.557	0.238, 0.073, 0.095–0.382, 0.001^*^, 0.577	0.312, 0.103, 0.108–0.515, 0.003^*^, 0.562	0.325, 0.109, 0.110–0.540, 0.003^*^, 0.544	0.209, 0.058, 0.096–0.323, <0.001^*^, 0.524
Light intensity (*Depression*)	0.295, 0.099, 0.100–0.491, 0.003^*^, 0.423	0.260, 0.083, 0.096–0.424, 0.002^*^, 0.405	0.329, 0.115, 0.103–0.555, 0.005^*^, 0.435	0.376, 0.117, 0.146–0.605, 0.001^*^, 0.464	0.201, 0.061, 0.081–0.320, 0.001^*^, 0.449
Light intensity (*Diabetes*)	0.076, 0.091, −0.104–0.257, 0.404, 0.620	0.085, 0.080, −0.072–0.243, 0.287, 0.600	0.065, 0.192, −0.137–0.266, 0.527, 0.630	0.018, 0.106, −0.190–0.227, 0.862, 0.654	0.170, 0.061, 0.051–0.290, 0.005^*^, 0.524
Light intensity (*Heart failure*)	0.222, 0.072, 0.080–0.365, 0.002^*^, 0.764	0.195, 0.067, 0.064–0.327, 0.004^*^, 0.712	0.238, 0.077, 0.086–0.390, 0.002^*^, 0.794	0.205, 0.080, 0.047–0.363, 0.011^*^, 0.799	0.190, 0.059, 0.074–0.307, 0.002^*^, 0.541
Light intensity (*Hyperlipidemia*)	0.145, 0.102, −0.056–0.346, 0.157, 0.518	0.136, 0.086, −0.034–0.305, 0.117, 0.506	0.132, 0.117, −0.099–0.363, 0.262, 0.534	0.143, 0.125, −0.103–0.390, 0.253, 0.508	0.163, 0.064, 0.036–0.290, 0.012^*^, 0.489
Light intensity (*Hypertension*)	0.063, 0.093, −0.120–0.246, 0.500, 0.645	0.102, 0.084, −0.064–0.267, 0.227, 0.573	0.024, 0.101, −0.174–0.223, 0.809, 0.683	−0.037, 0.104, −0.242–0.167, 0.719, 0.713	0.174, 0.063, 0.049–0.298, 0.006^*^, 0.479
Light intensity (*Stroke*)	0.155, 0.092, −0.025–0.335, 0.092, 0.598	0.195, 0.079, 0.038–0.351, 0.015^*^, 0.542	0.149, 0.105, −0.057–0.355, 0.155, 0.607	0.122, 0.107, −0.088–0.332, 0.254, 0.624	0.187, 0.060, 0.068–0.306, 0.002^*^, 0.517
Light intensity (*Obesity*)	0.284, 0.102, 0.083–0.485, 0.006^*^, 0.382	Data not available	Data not available	Data not available	Data not available
Light intensity (*All covariates*)	−0.004, 0.085, −0.170–0.163, 0.967, 0.815	−0.006, 0.075, 0.155–0.142, 0.933, 0.796	0.017, 0.093, −0.166–0.199, 0.857, 0.821	−0.068, −0.088, −0.241–0.106, 0.441, 0.861	0.174, 0.064, 0.049–0.300, 0.007^*^, 0.647

Linear mixed effects model data are provided for 10 chronic conditions that are established in the literature to impact risk of AD and/or AD incidence/prevalence. *p*-values are given for light pollution when considering the covariate. Non-significant *p-*values indicate those covariates that have a stronger influence on AD than light pollution. SE, standard error; CI, confidence interval; CKD, chronic kidney disease. *The bold indicate a significant *p* value.

### County analysis

**Figure 2:**
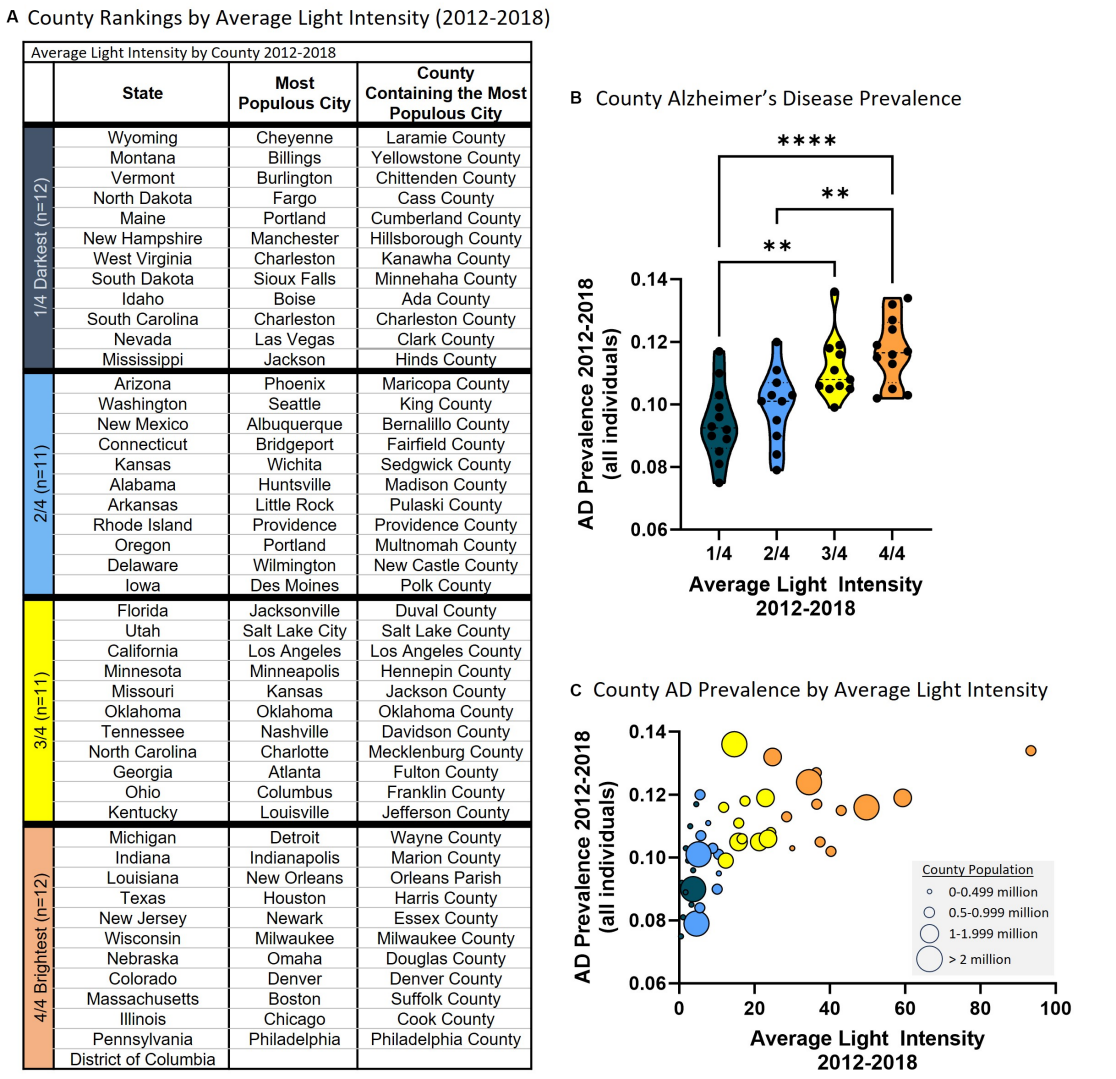
Higher average county nighttime light intensity is associated with higher county AD prevalence (2012–2018 average). **(A)** Average county nighttime light intensity rankings. 1/4 = darkest average nighttime light intensity through 4/4 = brightest average nighttime light intensity. **(B)** County AD prevalence by average county nighttime light intensity rankings. ANOVA revealed a statistically significant difference in AD prevalence between groups [*F*(3, 42) = 10.750, *p* < 0.001]. Multiple comparisons testing found that the counties with the lowest average light intensity had significantly different AD prevalence than counties with higher average nighttime light intensity: 1/4 vs. 3/4 (*p* = 0.003), 1/4 vs. 4/4 (*p* < 0.001), 2/4 vs. 4/4 (*p* = 0.002). ^**^p < 0.01, ^****^p < 0.0001. **(C)** Pearson correlation analysis between county AD prevalence and average county nighttime light intensity revealed a positive relationship [*r*(44) = 0.599, *p* < 0.001]. Circle size reflects county population.

Each state is heterogeneous including urban, suburban, and rural areas each with different exposures (e.g., dietary habits/patterns, activity level, air pollution, light pollution). Therefore, we examined the relationship between average nighttime light intensity and AD prevalence in counties since these are smaller in size and more homogeneous than the state level data. To do so, the largest city in each state was identified and the county in which this city resided was noted and average nighttime light intensity was determined for that county and compared to AD prevalence from Medicare Chronic Conditions data at the county level. Using this approach, data was retrieved for 45 counties and the District of Columbia. The data were subsequently clustered based on average nighttime light intensity representing those counties with the lowest average nighttime light intensity (i.e., 1/4, darkest) to the highest average nighttime light intensity (i.e., 4/4, brightest) (Figure 2A). Analysis revealed a statistically significant difference in AD prevalence between groups [*F*(3, 42) = 10.750, *p* < 0.001]. Multiple comparisons testing found that states with the darkest average nighttime light intensity were statistically different from states with brightest average nighttime light intensity (Figure 2B). Pearson correlation analysis similarly demonstrated a relationship between light intensity and AD prevalence [*r*(44) = 0.599, *p* < 0.001, Figure 2C]. This relationship also existed when examining those over the age of 65 [ANOVA: *F*(3, 42) = 10.840, *p* < 0.001; Correlation: *r*(44) = 0.612, *p* < 0.001] and those under the age of 65 [ANOVA: *F*(3, 42) = 8.424, *p* < 0.001; Correlation: *r*(44) = 0.514, *p* < 0.001] (Supplementary Figure S2).

A linear mixed effects model was applied to examine the overall effect of average light intensity on AD prevalence (from 2012 to 2018) considering within-county correlation due to repeated measures. When examining all individuals, average nighttime light intensity was significantly association with AD prevalence (regression coefficient = 0.040, SE = 0.003, 95% CI = 0.034–0.047, *p* < 0.001, *R*^2^ = 0.304) including in those over the age of 65 (regression coefficient = 0.052, SE = 0.004, 95% CI = 0.043–0.060, *p* < 0.001, *R*^2^ = 0.304) and under the age of 65 (regression coefficient = 0.019, SE = 0.002, 95% CI = 0.015–0.023, *p* < 0.001, *R*^2^ = 0.480). In summary, the results observed on the state level are recapitulated on the county level bolstering support for the positive association between AD prevalence and nighttime light exposure.

## Discussion

### Average light intensity is associated with AD prevalence

The analyses reveal that greater average nighttime light intensity (i.e., light pollution) was associated with higher AD prevalence. This was true for 2012–2018 average and each year examined individually, and in those over and under the age of 65 (i.e., 65+, <65), in both sexes, and in each race (except Asian Pacific Island which may be related to power). This finding was observed when examining data on the state level as well as on the county level.

Average nighttime light intensity was more strongly associated with AD prevalence than some diseases and conditions reported or suspected to be risk factors for AD including alcohol abuse (Anstey et al., 2009; Andrews et al., 2020; Nallapu et al., 2023; Wang et al., 2023), chronic kidney disease (Zhang et al., 2020; Tang et al., 2022), depression (Saiz-Vazquez et al., 2021), heart failure (Qiu et al., 2006; Cermakova et al., 2015), and obesity (Alford et al., 2018; Al-Kuraishy et al., 2023). However, other covariates were more strongly associated with AD than average nighttime light intensity including atrial fibrillation (Giannone et al., 2022), diabetes, hyperlipidemia, hypertension, and stoke. This finding is perhaps not surprising as the effects of nighttime light exposure would be expected to be more subtle than factors robustly associated with AD. However, in individuals under the age of 65, nighttime light intensity was associated with AD prevalence to a greater degree than all other disease risk factors, suggesting that younger individuals may be particularly sensitive to the detrimental effects of light at night. While it is challenging to speculate about why those under the age of 65 would be particularly vulnerable to the effects of nighttime light exposure, literature demonstrates that there are individual differences in light sensitivity (Chellappa, 2021). Indeed, APOE genotype—a factor influencing early onset AD—impacts response to biological stressors (e.g., oxidative stress) and this could account for the increased vulnerability to the effects of nighttime light exposure (Jofre-Monseny et al., 2008; Dose et al., 2016). However, more research on how light at night may influence AD is needed.

### Nighttime light exposure: an important part of the exposome

Dementia is not a modern phenomenon. Dementia is mentioned by Pythagoras, Hippocrates, Plato, and Shakespeare (Yang et al., 2016) and yet since the development of diagnostic criteria and AD incidence and prevalence have been reported, rates of AD have risen dramatically. This increase is attributed to factors like the aging population, emergence of lifestyle related diseases like obesity, high blood pressure, and diabetes, and a myriad of environmental factors related to urbanization and the industrial revolution. One such example is air pollution which is associated with cognitive decline (Franz et al., 2023), dementia (Gong et al., 2023), faster cognitive decline in those with AD (Lee et al., 2023), and incident neurodegenerative disease (Zhu et al., 2023). Exposure to light at night could similarly be impacting the development or progression of dementia and neurodegenerative disease.

To illustrate this possibility, we can examine Amish communities (who intentionally limit their use of modern technology and electricity and may consequently be exposed to less light at night). Amish communities have a lower prevalence of cognitive impairment in individuals of advanced age compared to the general population (Pericak-Vance et al., 1996; Johnson et al., 1997; Holder and Warren, 1998). Numerous factors other than light exposure may contribute to this phenomenon (e.g., emphasis on family/community, traditional farming, genetics), but that being said, exposure of Drosophila to dim light during the dark period promotes neurodegeneration (Kim et al., 2018), and data from mice demonstrate that dim light during the dark period alters neuronal dendrites in AD-relevant brain regions (cortex, hippocampus) (Delorme et al., 2022), and long-term exposure to outdoor light at night is associated with increased risk of cognitive impairment in humans (Chen et al., 2022), and there is a positive correlation between light pollution and Parkinson’s disease (another neurodegenerative disease) (Romeo et al., 2013). Additional studies carefully evaluating indoor and outdoor light exposure and mechanistic evaluations are needed to fully understand the impact of nighttime light exposure and light pollution on AD.

The analyses in this report encompass years 2012 through 2018 during which time there was a trend for states to have a decrease in nighttime light (2012 = 100,000, 2018 = 96.890, *p* = 0.054). The pandemic further decreased nighttime light exposure which was at the lowest level in 2020; however, despite the fact that 17 states have legislation designed to reduce light pollution nighttime light was the highest in 2022 (latest data available) (2012 = 100,000, 2022 = 103,500, *p* = 0.007) (Supplementary Figure S3). Given the association between nighttime light exposure and AD prevalence the increased nighttime light could potentially impact AD incidence and prevalence.

### Potential mechanisms by which light pollution may promote Alzheimer’s disease

There are multiple mechanisms by which exposure to light at night may influence AD development or progression. First, there is a plethora of literature demonstrating that exposure to light at night is associated with sleep disruption. Sleep disruption can activate microglia and astrocytes, promote inflammation, negatively alter the clearance of amyloid beta (potentially via the glymphatic system), is associated with loss of neurons in the cortex, hippocampus, and locus coeruleus, and hippocampal atrophy (Kang et al., 2009; Xia et al., 2017; Atrooz et al., 2019; Reddy and van der Werf, 2020; Xiao et al., 2020; Owen et al., 2021; Zamore and Veasey, 2022). A meta-analysis extrapolating from 27 observational studies reports that individuals with sleep problems have a higher risk of cognitive impairment and AD, compared to individuals without sleep problems (Bubu et al., 2017). These observations share many features that are common with AD. Second, exposure to light at night may disrupt circadian rhythms (Raap et al., 2015) and sleep is one output of circadian rhythms perhaps suggesting an upstream disruption in circadian rhythms. Circadian rhythm disruption is widely reported by our group and others to have detrimental effects on health. Changes in circadian rhythms often precede symptoms of AD in humans (Colwell, 2021) and it is interesting to consider that exposure to light at night (via circadian disruption) may contribute to AD pathogenesis (Musiek, 2015; Musiek, 2017). Additionally, it is noteworthy that disruption of circadian rhythms is associated with increased risk of diseases that are risk factors for AD including obesity, diabetes, and depression, just to name a few (Fishbein et al., 2021). Lastly, on the biochemical level, exposure of mice to dim light increases the production of the pro-inflammatory cytokine interleukin 1β (IL-1β) (Walker et al., 2020), which is a feature associated with AD and exposure of mice to dim light during the dark period decreases levels of the neurotrophic factor brain derived neurotrophic factor (BDNF) in a brain region relevant for AD (i.e., the hippocampus) (Walker et al., 2020). This finding is intriguing as our group has demonstrated that low levels of BDNF are a feature that precede cognitive impairment (Voigt et al., 2021). Taken together there are numerous mechanisms by which exposure to light at night can impact AD, the relative contributions of these mechanisms will require additional investigation.

### Caveats

There are limitations associated with this study related to the curation of AD prevalence and light exposure data. First, the Medicare data are limited to those enrolled in Medicare Part A and Part B. Those with Medicare Advantage, only Part A, or only Part B are excluded; thus, the data presented in this report are not comprehensive (although Medicare data are reported to be reasonably reliable) (Grodstein et al., 2022). Second, the Medicare data report current residences of individuals which may not reflect life-long residence (important to understand causality). Third, this study evaluated AD prevalence and not incidence. Fourth, this study used state and county average light intensity data from satellites. No indoor light data were available although indoor light exposure (e.g., televisions, computers, phones) is critically important and should be evaluated in future studies. However, living in an area with more intense outdoor light at night is associated with shorter sleep duration, increased daytime sleepiness, and dissatisfaction with sleep quality suggesting that outdoor light can meaningfully impact biology providing validity to the notion that outdoor light exposure is important (Ohayon and Milesi, 2016; Xiao et al., 2020). However, the totality of outdoor and indoor nighttime light exposure is important to consider to fully understand the impact of nighttime light on AD.

## Conclusion

Many (but not all) studies suggest that AD incidence has declined during the last decade (Rocca et al., 2011; Schrijvers et al., 2012; Matthews et al., 2013, 2016; Satizabal et al., 2016; Ahmadi-Abhari et al., 2017; Cerasuolo et al., 2017; Derby et al., 2017; Langa et al., 2017; Wu et al., 2017; Sullivan et al., 2019; Wolters et al., 2020). This is thought to be attributed to better treatment of AD-associated risk factors (e.g., hypertension, diabetes), greater educational attainment, and perhaps greater awareness of factors that contribute to AD (e.g., diet) (Hudomiet et al., 2022). Data from this study suggest that reduction of these major factors may cause environmental factors like nighttime light exposure to have a substantial influence on AD pathogenesis in the future. Additionally, AD prevalence is expected to continue to increase due to increased life expectancy (Kawas et al., 2000; Fitten et al., 2001; Guerreiro and Bras, 2015; Mayeda et al., 2016, 2017; Ajrouch et al., 2017; Matthews et al., 2019; Grodstein et al., 2022) and it is of value to understand how exposure to light at night influences AD progression. While data from preclinical studies and the current study suggest exposure to light at night may influence AD, additional studies evaluating clinical and population health are needed.

## Methods

### Sex as a biological variable

De-identified data were obtained from Medicare reports of disease prevalence. All available data were included inclusive of both sexes.

### Alzheimer’s disease prevalence and co-variate data acquisition

#### Medicare data

Medicare reports of disease prevalence from 2012 to 2018 were used. The data were obtained from the Office of Enterprise Data and Analytics, within the Centers for Medicare and Medicaid Services (CMS) which develops analytics examining chronic conditions among Medicare fee-for-service beneficiaries. Medicare is Federal a health insurance program for individuals over the age of 65, persons 65 years and under with certain disabilities, and individuals of any age with end-stage renal disease in the United States.

The data used in the chronic conditions report are based on CMS administrative enrollment and claims data for Medicare beneficiaries enrolled in the fee-for-service program. The data are available from the CMS Chronic Conditions Data Warehouse (CCW).^1^ The chronic conditions public use files report prevalence in the Medicare beneficiary population limited to fee-for-service beneficiaries. Excluded populations include: (1) Medicare beneficiaries with any Medicare Advantage enrollment during the year and (2) beneficiaries who were enrolled in Part A only or Part B only. In 2018, exclusions accounted for approximately 44.9% of the total population. Beneficiaries who died during the year were included up to the date of death if other inclusion criteria were met.

The CMS CCW database includes pre-defined indicators for chronic conditions and mental health conditions (details on these conditions is available at: www.ccwdata.org). A Medicare beneficiary is considered to have a chronic condition if a claim indicates that the beneficiary received a service or treatment for the specific condition. Chronic conditions are identified by diagnosis codes on the Medicare claims. Services prior to October 2015 used International Classification of Diseases version 9 (ICD-9-CM) codes, chronic conditions identified in or after October 2015 used version 10 (ICD-10-CM-December). Beneficiaries may have more than one chronic condition. Estimates of the prevalence of Chronic Conditions may vary from other sources, as estimates of Chronic Conditions will be influenced by the number and type of conditions that are used. Geographic variation in the prevalence estimates of chronic conditions can be affected by using diagnoses on administrative claims to infer the presence of a condition. Variability in coding diagnoses can lead to both the under and over diagnosis of specific conditions and affect estimates of chronic conditions.

Estimates are measures of overall magnitude of chronic conditions in the Medicare population for national, state, and county levels. Data are presented based upon the beneficiary’s residence, rather than where care was received. Prevalence estimates are not age or sex adjusted nor are they adjusted for beneficiary characteristics across geographic variability. There is evidence that regional variation in care is associated with the supply of health care resources, which can affect prevalence estimates; since in places where more healthcare resources are available, the likelihood that diagnoses will be identified may be increased. A recent assessment found that Medicare claims perform reasonably well in identifying dementia (Grodstein et al., 2022).

#### Center for disease control and prevention data

Obesity data were obtained from the Center for Disease Control and Prevention (CDC). In 1984 the CDC initiated the state-based Behavioral Risk Score Surveillance System (BRFSS),^2^ a cross-sectional telephone survey that state health departments conduct monthly with a standardized questionnaire and technical and methodological assistance from CDC. BRFSS is used to collect prevalence data among adult residents in the United States regarding their risk behaviors and preventive health practices that can affect their health status. Respondent data are forwarded to CDC to be aggregated for each state. Which reports self-reported adult obesity prevalence by race, ethnicity, and location (methodological details at: http://www.cdc.gov/brfss/factsheets/pdf/DBS_BRFSS_survey.pdf). Data can be found at: https://data.cdc.gov/Behavioral-Risk-Factors/BRFSS-Table-of-Overweight-and-Obesity-BMI-/fqb7-mgjf.

### Nighttime light exposure data acquisition

Nighttime light exposure data was generated by the www.lightpollutionmap.info platform (Jurij Stare)^3^ which maps radiance data from NASA’s VIIRS/NPP Lunar BRDF-Adjusted Nighttime Lights Yearly composites (AllAngle_Composite_Snow_Free).

The Day/Night Band (DNB) sensor of the Visible Infrared Imaging Radiometer Suite (VIRRS), on board the Suomi-National Polar-orbiting Partnership (S-NPP) and Joint Polar Satellite System (JPSS) satellite platforms, provide daily measurements of nocturnal visible and near-infrared (NIR) light. NASA developed a suite of products of nighttime lights (NTL) applications including NASA’s Black Marble product suite (VNP46/VJ146). NASA’s Black Marble nighttime lights product, at 15 arc-second spatial resolution, is available from January 2012-present with data from the VIIRS DNB sensor. The VNP46/VJ146 product suite includes the daily at-sensor top of atmosphere (TOA) nighttime lights (NTL) product (VNP46A1/VJ146A1), daily moonlight-adjusted nighttime lights product (VNP46A2/VJ146A2), monthly moonlight-adjusted nighttime lights product (VNP46A3/VJ146A3), and yearly moonlight-adjusted nighttime lights product (VNP46A4/VJ146A4). To remove residual background noise, NASA’s VIIRS/NPP Lunar BRDF-Adjusted NTL composite values with radiances less than 0.5 nW.cm-2.sr-1 are set to zero. The retrieval algorithm, developed and implemented for routine global processing at NASA’s Land Science Investigator-led Processing System (SIPS), utilizes all high-quality, cloud-free, atmospheric-, terrain-, vegetation-, snow-, lunar-, and stray light-corrected radiance to estimate daily nighttime lights and other intrinsic surface optical properties. Details about algorithms, operational processing, evaluation, and validation are found in the user guide (Roman et al., 2021). Data for each year include from January 1st through December 31.

VIRRS data were overlaid on a map and areas of interest (i.e., states, counties) were outlined in triplicate (three independent measurements) and values were averaged to develop the average nighttime light intensity for each area of interest. Radiance data are provided as 10^−9^ W/cm^2 *^ sr. World Atlas images were developed using Falchi et al. (2016, 2017).

### Statistics

Average nighttime light intensity data were generated for each state (excluding Alaska and Hawaii) or county and data were analyzed as an average of 2012–2018 as well as each year independently for each state. States were divided into five groups and county data divided into four groups according to average nighttime light intensity and an ANOVA was conducted followed by a *post hoc* Tukey for pairwise comparisons (GraphPad Prism 10.0.2) to compare AD prevalence among the groups. Correlation analysis was also conducted with state and county data to evaluate the relationship between AD prevalence and average nighttime light exposure (GraphPad Prism 10.0.2).

A linear mixed model was then applied to examine the relationship with all data together taking account of within-state or within-county correlation due to repeated measures. The state and county assessments included age, sex, and race in the model. For the state data, biological covariates (atrial fibrillation, chronic kidney disease, depression, diabetes, heart failure, hyperlipidemia, hypertension, obesity, and stroke) were considered and subgroup analyses were performed including: age (65+, <65), sex (men, women), and race (Asian Pacific Islander, Hispanic, Native American, Non-Hispanic Black, Non-Hispanic White). Analyses were conducted using SAS (v 9.4).

## One-sentence summary

There is a positive association between Alzheimer’s disease prevalence and average outdoor nighttime light intensity in the United States.

## Data availability statement

The original contributions presented in the study are included in the article/Supplementary material, further inquiries can be directed to the corresponding author.

## Ethics statement

Ethical approval was not required for the study involving humans in accordance with the local legislation and institutional requirements. Written informed consent to participate in this study was not required from the participants or the participants' legal guardians/next of kin in accordance with the national legislation and the institutional requirements.

## Author contributions

RV: Conceptualization, Data curation, Funding acquisition, Investigation, Methodology, Project administration, Resources, Supervision, Visualization, Writing – original draft, Writing – review & editing. BO: Formal analysis, Investigation, Methodology, Writing – original draft, Writing – review & editing. AK: Funding acquisition, Resources, Writing – review & editing.

## Footnotes

^1^ www.ccwdata.org

^2^ https://www.cdc.gov/brfss/

^3^ www.lightpollutionmap.info
