# Supplementary material for: Outdoor nighttime light exposure (light pollution) is associated with Alzheimer’s disease
Source: Front Neurosci. 2024 Sep 6;18:1378498. doi: 10.3389/fnins.2024.1378498 (PMC11412842; doi:10.3389/fnins.2024.1378498)
Supplement: Supplementary file 1 [file Table_1.docx]

| **Supplementary Table 1.** | | | |
| --- | --- | --- | --- |
| Year | State Groupings (relevant for ANOVA analysis) | ANOVA Analysis | Correlation Analysis |
| 2012 | Montana, Wyoming, South Dakota, Idaho, New Mexico, Maine, Oregon, Vermont, Utah, Nevada | *ALL INDIVIDUALS*  ANOVA: F(4,43)=13.12, ***p<0.001****  *65+*  ANOVA: F(4,43)=15.64, ***p<0.001****  *<65*  ANOVA: F(4,43)=7.438, ***p<0.001**** | *ALL INDIVIDUALS*  Correlation: r(46)=0.611, ***p<0.001****  *65+*  Correlation: r(46)=0.560, ***p<0.001****  *<65*  Correlation: r(46)=0.595, ***p<0.001**** |
|  | Nebraska, North Dakota, Colorado, Kansas, Arizona, Minnesota, Washington, Texas, New Hampshire, Iowa |  |  |
|  | Arkansas, West Virginia, Oklahoma, Wisconsin, Michigan, Mississippi, Missouri, California |  |  |
|  | Alabama, Kentucky, Louisiana, New York, Virginia, Pennsylvania, North Carolina, South Carolina, Georgina, Tennessee |  |  |
|  | Indiana, Illinois, Florida, Ohio, Massachusetts, Connecticut, Maryland, Delaware, Rhode Island, New Jersey |  |  |
| 2013 | Montana, Wyoming, Idaho, South Dakota, Maine, New Mexico, Vermont, Oregon, Utah, Nevada | *ALL INDIVIDUALS*  ANOVA: F(4,43)=11.65, ***p<0.001****  *65+*  ANOVA: F(4,43)=12.37, ***p<0.001****  *<65*  ANOVA: F(4,43)=6.686, ***p=0.0003**** | *ALL INDIVIDUALS*  Correlation: r(46)=0.574, ***p<0.001****  *65+*  Correlation: r(46)=0.519, ***p<0.001****  *<65*  Correlation: r(46)=0.574, ***p<0.001**** |
|  | Nebraska, North Dakota, Colorado, Kansas, Arizona, Minnesota, Washington, Texas, Arkansas, Iowa |  |  |
|  | New Hampshire, West Virginia, Oklahoma, Wisconsin, Michigan, Missouri, California |  |  |
|  | Alabama, Kentucky, Louisiana, New York, Virginia, North Carolina, South Carolina, Pennsylvania, Georgia, Tennessee, |  |  |
|  | Indiana, Illinois, Florida, Ohio, Massachusetts, Connecticut, Maryland, Delaware, Rhode Island, New Jersey |  |  |
| 2014 | Montana, Wyoming, Idaho, South Dakota, New Mexico, Maine, Oregon, Vermont, Utah, Nevada | *ALL INDIVIDUALS*  ANOVA: F(4,43)=12.51, ***p<0.001****  *65+*  ANOVA: F(4,43)=12.79, ***p<0.001****  *<65*  ANOVA: F(4,43)=6.738, ***p<0.001**** | *ALL INDIVIDUALS*  Correlation: r(46)=0.569, ***p<0.001****  *65+*  Correlation: r(46)=0.514, ***p<0.001****  *<65*  Correlation: r(46)=0.541, ***p<0.001**** |
|  | Nebraska, Colorado, Kansas, North Dakota, Arizona, Minnesota, Washington, Texas, Iowa, Arkansas |  |  |
|  | New Hampshire, Oklahoma, West Virginia, Wisconsin, Michigan, Mississippi, Missouri, California |  |  |
|  | Alabama, Kentucky, Louisiana, New York, Virginia, North Carolina, Pennsylvania, South Carolina, Georgina, Tennessee |  |  |
|  | Indiana, Illinois, Florida, Ohio, Massachusetts, Connecticut, Maryland, Delaware, Rhode Island |  |  |
| 2015 | Montana, Wyoming, Idaho, South Dakota, Maine, Vermont, New Mexico, Oregon, Utah, Nevada | *ALL INDIVIDUALS*  ANOVA: F(4,43)=13.38, ***p<0.001****  *65+*  ANOVA: F(4,43)=14.81, ***p<0.001****  *<65*  ANOVA: F(4,43)=6.410, ***p<0.001**** | *ALL INDIVIDUALS*  Correlation: r(46)=0.554, ***p<0.001****  *65+*  Correlation: r(46)=0.491, ***p<0.001****  *<65*  Correlation: r(46)=0.544, ***p<0.001**** |
|  | Nebraska, Colorado, Kansas, North Dakota, Arizona, Minnesota, Washington, Iowa, Texas, New Hampshire |  |  |
|  | Arkansas, West Virginia, Oklahoma, Wisconsin, Michigan, Missouri California |  |  |
|  | Alabama, Kentucky, Louisiana, Virginia, Pennsylvania, North Carolina, South Carolina, Georgia Tennessee |  |  |
|  | Indiana, Illinois, Florida, Ohio, Massachusetts, Connecticut, Maryland, Delaware, Rhode Island, New Jersey |  |  |
| 2016 | Montana, Wyoming, South Dakota, Idaho, Maine, New Mexico, Vermont, Oregon, Utah, Nevada | ALL INDIVIDUALS  ANOVA: F(4,43)=12.29, ***p<0.001****  *65+*  ANOVA: F(4,43)=13.44, ***p<0.001****  *<65*  ANOVA: F(4,43)=7.090, ***p<0.001**** | ALL INDIVIDUALS  Correlation: r(46)=0.527, ***p<0.001****  *65+*  Correlation: r(46)=0.456, ***p=0.001****  *<65*  Correlation: r(46)=0.544, ***p<0.001**** |
|  | Nebraska, North Dakota, Kansas, Colorado, Minnesota, Arizona, Washington, Iowa, New Hampshire, Texas |  |  |
|  | Arkansas, West Virginia, Oklahoma, Wisconsin, Michigan, Mississippi, Missouri, California |  |  |
|  | Alabama, Kentucky, Louisiana, New York, Virginia, North Carolina, Pennsylvania, South Carolina, Georgia, Tennessee |  |  |
|  | Indiana, Illinois, Florida, Ohio, Massachusetts, Connecticut, Maryland, Delaware, Rhode Island, New Jersey |  |  |
| 2017 | Montana, Wyoming, South Dakota, Idaho, Maine, New Mexico, Oregon, Vermont, Utah, Nevada | *ALL INDIVIDUALS*  ANOVA: F(4,43)=11.69, ***p<0.001***  *65+*  ANOVA: F(4,43)=12.05, ***p<0.001***  *<65*  ANOVA: F(4,43)=6.459, ***p<0.001**** | *ALL INDIVIDUALS*  Correlation: r(46)=0.511, ***p<0.001****  *65+*  Correlation: r(46)=0.427, ***p=0.003****  *<65*  Correlation: r(46)=0.506, ***p<0.001**** |
|  | Nebraska, Kansas, Colorado, North Dakota, Minnesota, Arizona, Washington, Iowa, Texas, New Hampshire |  |  |
|  | Arkansas, West Virginia, Oklahoma, Wisconsin, Michigan, Mississippi, Missouri, Kentucky |  |  |
|  | California, Alabama, Louisiana, New York, Virginia, North Carolina, Pennsylvania, Georgia, South Carolina, Tennessee |  |  |
|  | Indiana, Illinois, Florida, Ohio, Massachusetts, Connecticut, Maryland, Delaware, Rhode Island, New Jersey |  |  |
| 2018 | Montana, Wyoming, south Dakota, Idaho, Maine, Vermont, Oregon, New Mexico, Utah, Nevada | *ALL INDIVIDUALS*  ANOVA: F(4,43)=9.413, ***p<0.001****  *65+*  ANOVA: F(4,43)=8.681, ***p<0.001****  *<65*  ANOVA: F(4,43)=7.153, ***p<0.001**** | *ALL INDIVIDUALS*  Correlation: r(46)=0.500, ***p<0.001****  *65+*  Correlation: r(46)=0.418, ***p=0.003****  *<65*  Correlation: r(46)=0.514, ***p<0.001**** |
|  | Nebraska, Colorado, Kansas, Minnesota, Arizona, Washington, Iowa, North Dakota, Texas, Arkansas |  |  |
|  | New Hampshire, West Virginia, Oklahoma, Wisconsin, Mississippi, Michigan, Missouri, Alabama |  |  |
|  | Kentucky, California, Louisiana, New York, Virginia Georgia, north Carolina, Pennsylvania, Tennessee, south Carolina |  |  |
|  | Indiana, Illinois, Florida, Ohio, Massachusetts, Connecticut Maryland Delaware, Rhode Island, New jersey |  |  |
| **Table S1: Analysis of State AD prevalence and average nighttime light intensity from 2012-2018.**  Each year (2012-2018) was individually assessed for the relationship between AD prevalence and average nighttime light intensity. A positive relationship was observed between AD prevalence and nighttime light intensity for each year. States were grouped according to highest to lowest average nighttime light intensity and these data were analyzed by analysis of variance (ANOVA). Results indicate a significant relationship between AD prevalence and light pollution). Linear correlation analysis was conducted between AD prevalence and average nighttime light intensity which demonstrated a significant positive correlation. | | | |
