# Supplementary figures and images for: Outdoor nighttime light exposure (light pollution) is associated with Alzheimer’s disease
Source: Front Neurosci. 2024 Sep 6;18:1378498. doi: 10.3389/fnins.2024.1378498 (PMC11412842; doi:10.3389/fnins.2024.1378498)

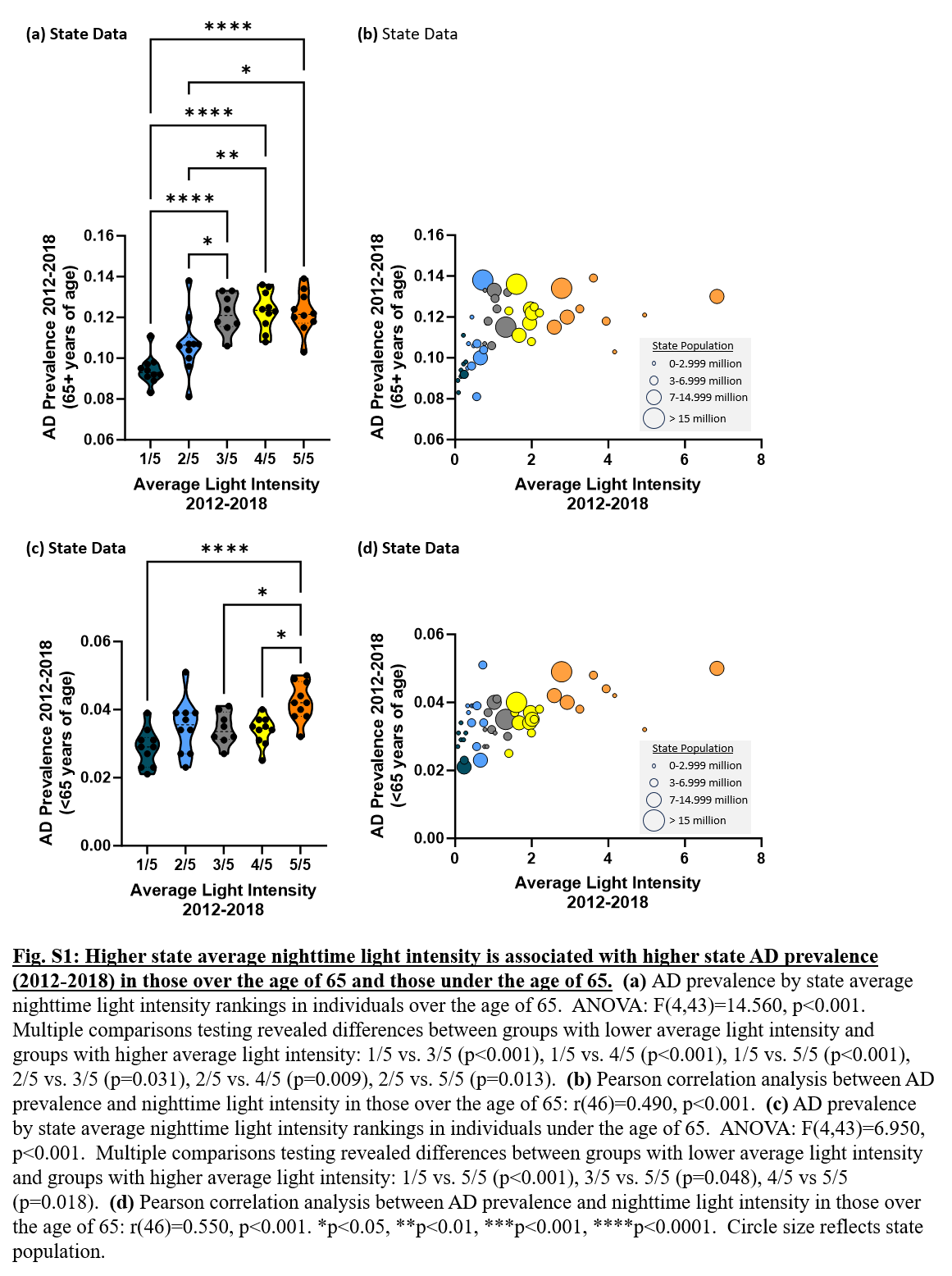

Supplement: Supplementary file 3 [file Image_1.tif]

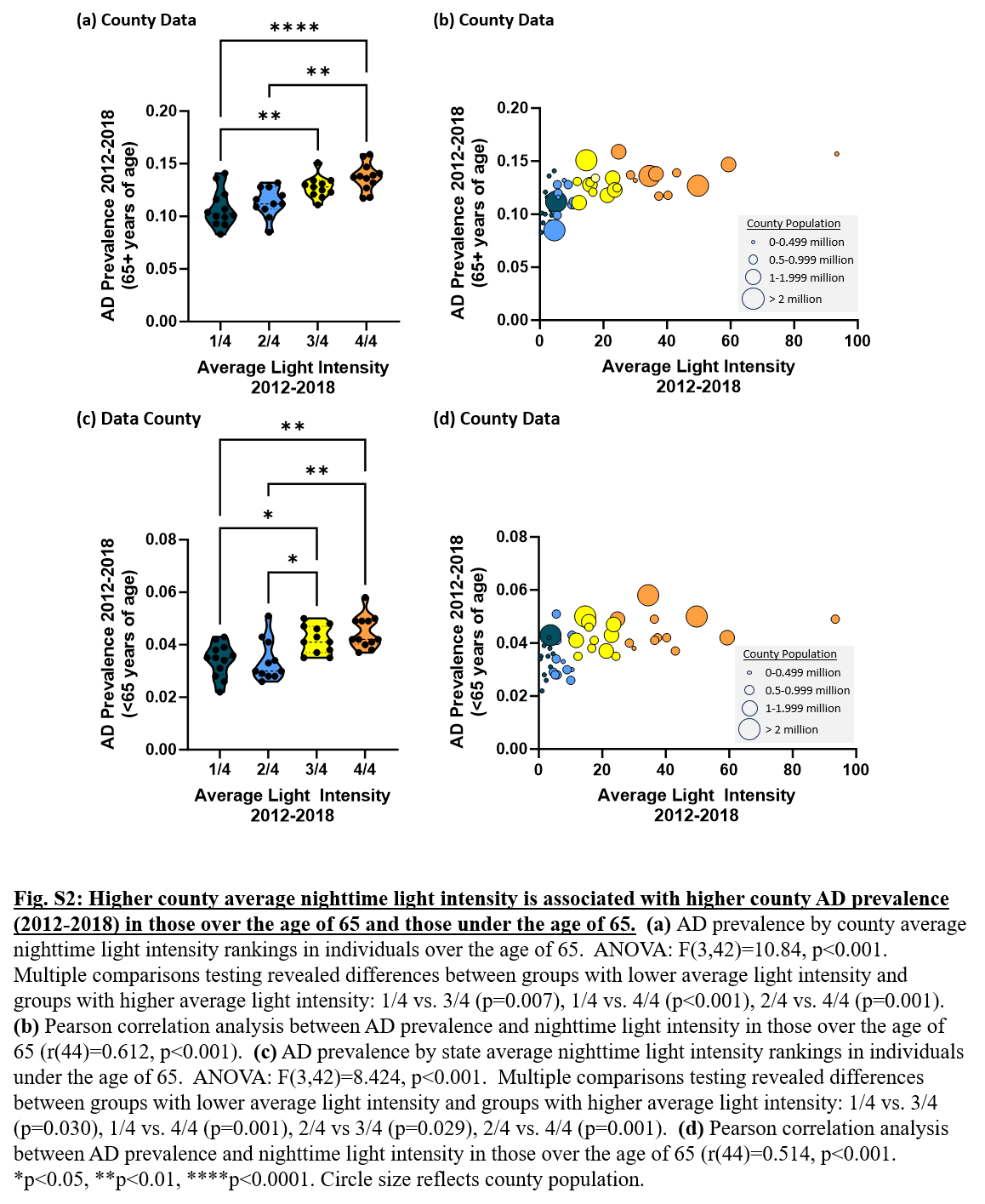

Supplement: Supplementary file 4 [file Image_2.tif]

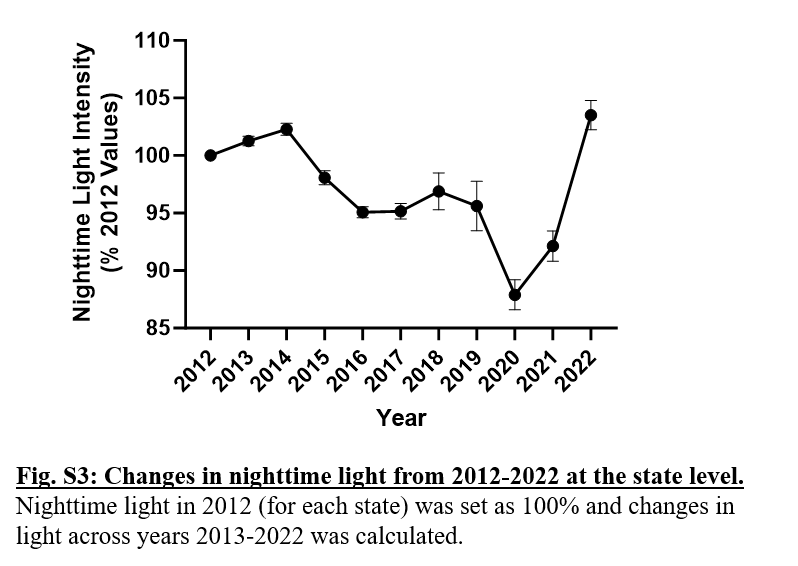

Supplement: Supplementary file 5 [file Image_3.tif]
